# Supplementary material for: LEM-3 is a midbody-tethered DNA nuclease that resolves chromatin bridges during late mitosis
Source: Nat Commun. 2018 Feb 20;9:728. doi: 10.1038/s41467-018-03135-w (PMC5820297; doi:10.1038/s41467-018-03135-w)
Supplement: Supplementary file 1 — Supplementary Information [file 41467_2018_3135_MOESM1_ESM.pdf]

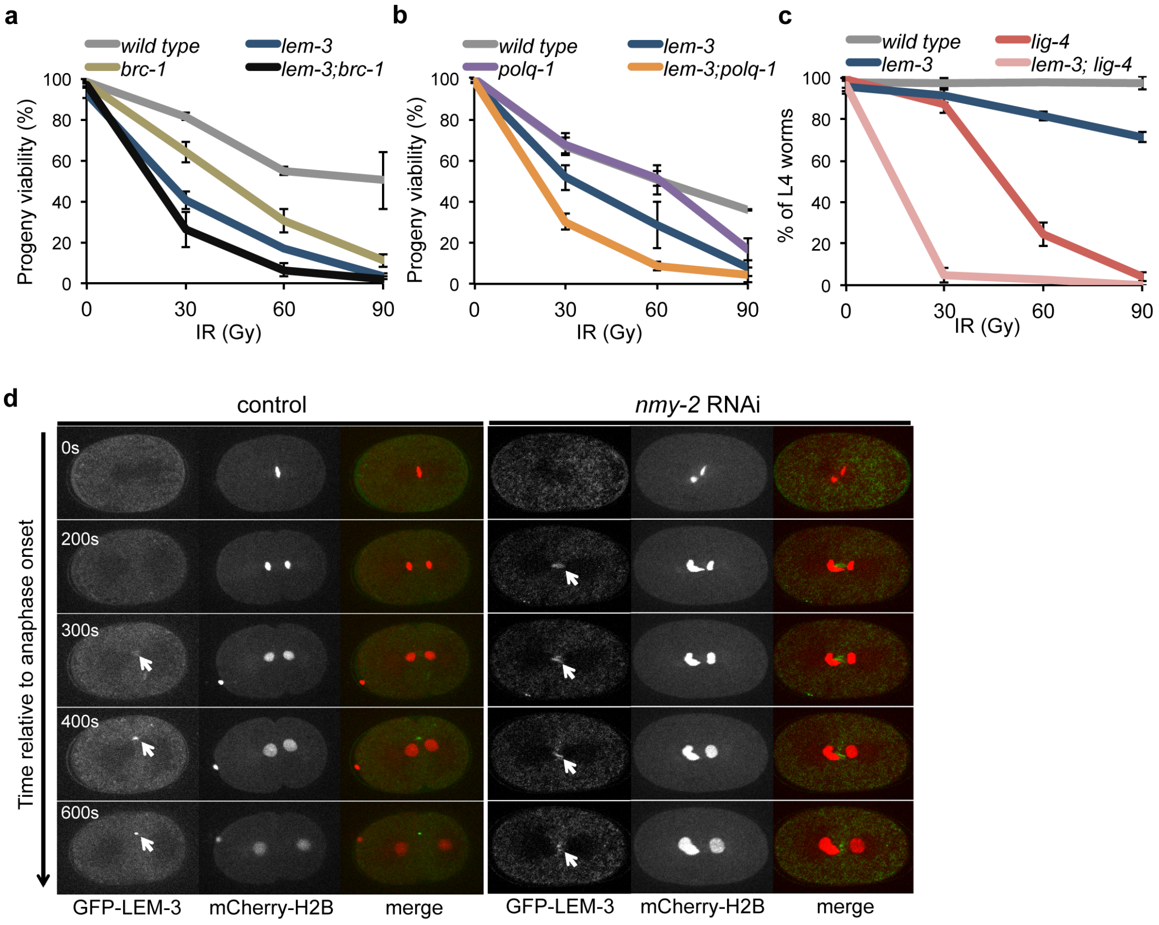

Supplementary Figure 1. (a and b) Sensitivity of *lem-3* single and double mutants to IR. Young nematodes were treated with ionizing radiation. After 24 hours of recovery, nematodes were transferred onto new plates and allowed to lay eggs for 6-8 hours. Progeny viability in % was determined by counting number of viable eggs/total number of eggs laid. (c) Late stage embryos were irradiated and the proportion of animals researching L4 stage was scored. This assay accounts for defect in end joining a pathway that predominantly acts in *C. elegans* somatic tissues. Error bars represent standard deviation of the mean. (d) Midbody localization of LEM-3 is independent on the contractile ring formation. Depletion of the contractile ring component NMY-2 by *nmy-2* RNAi didn't affect the recruitment of LEM-3 to the midbody. Images were taken from a time-lapse recording of embryos expressing GFP-LEM-3 and mCherry-H2B from the anaphase onset of the first mitotic division. Times are relative to anaphase onset.

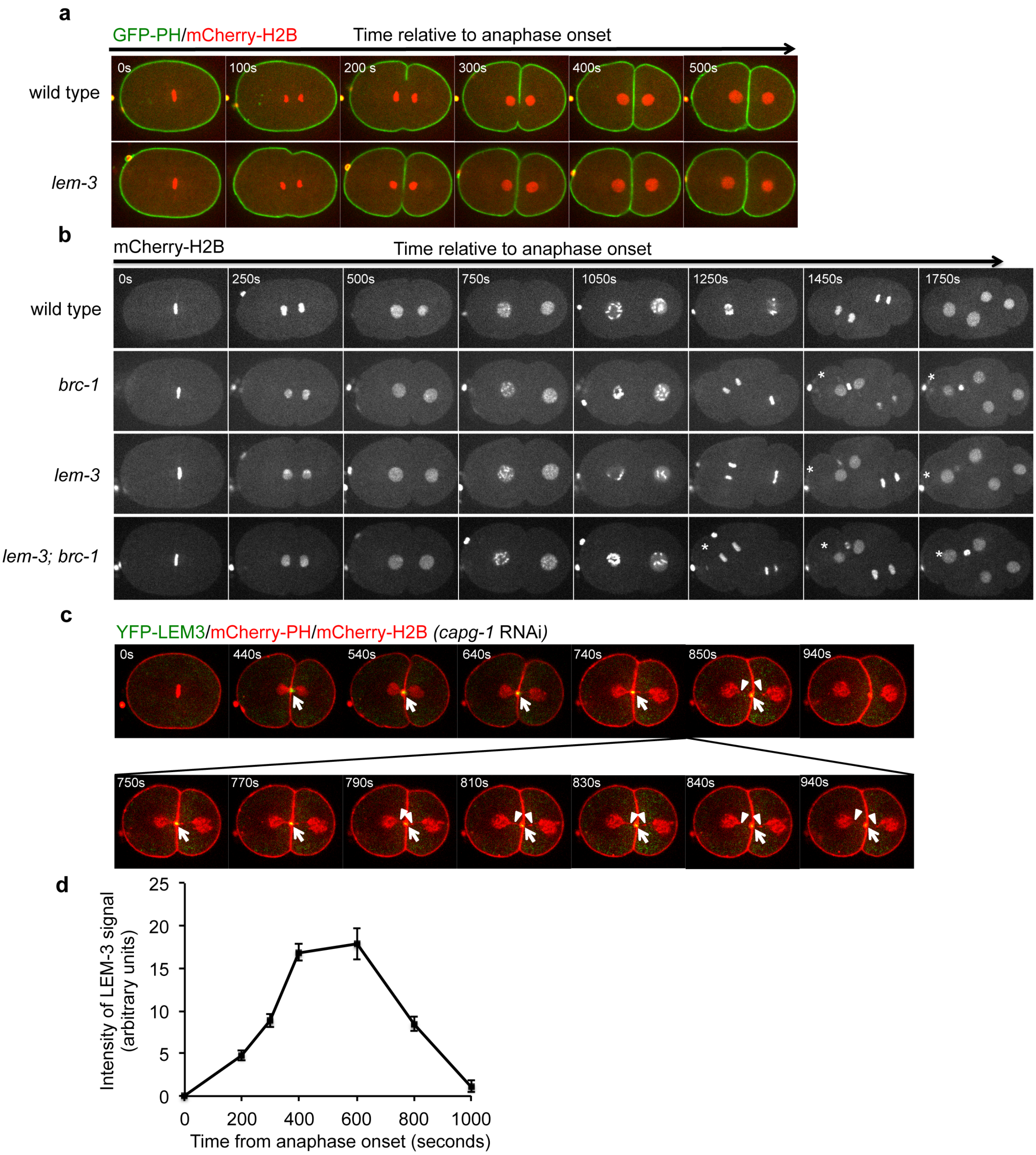

Supplementary Figure 2. Resolution of chromatin bridges by LEM-3. (a) Chromosome segregation in wild type and *lem-3* mutant embryos. Images were taken from embryos expressing mCherry-H2B and GFP-PH. (b) *C. elegans* of the indicated genotypes were dissected and one cell stage embryos were used for recording. Images were taken from time-lapse recordings of embryos expressing mCherry-H2B. Times are relative to anaphase onset of the first division. (c) Localization of LEM-3 before and after resolution of chromatin bridges induced by *capg-1* RNAi. Arrows indicate YFP-LEM-3. Arrowheads indicate retracting chromatin bridges. (d) Quantification of intensity of LEM-3 signal at the midbody during chromatin bridge resolution.

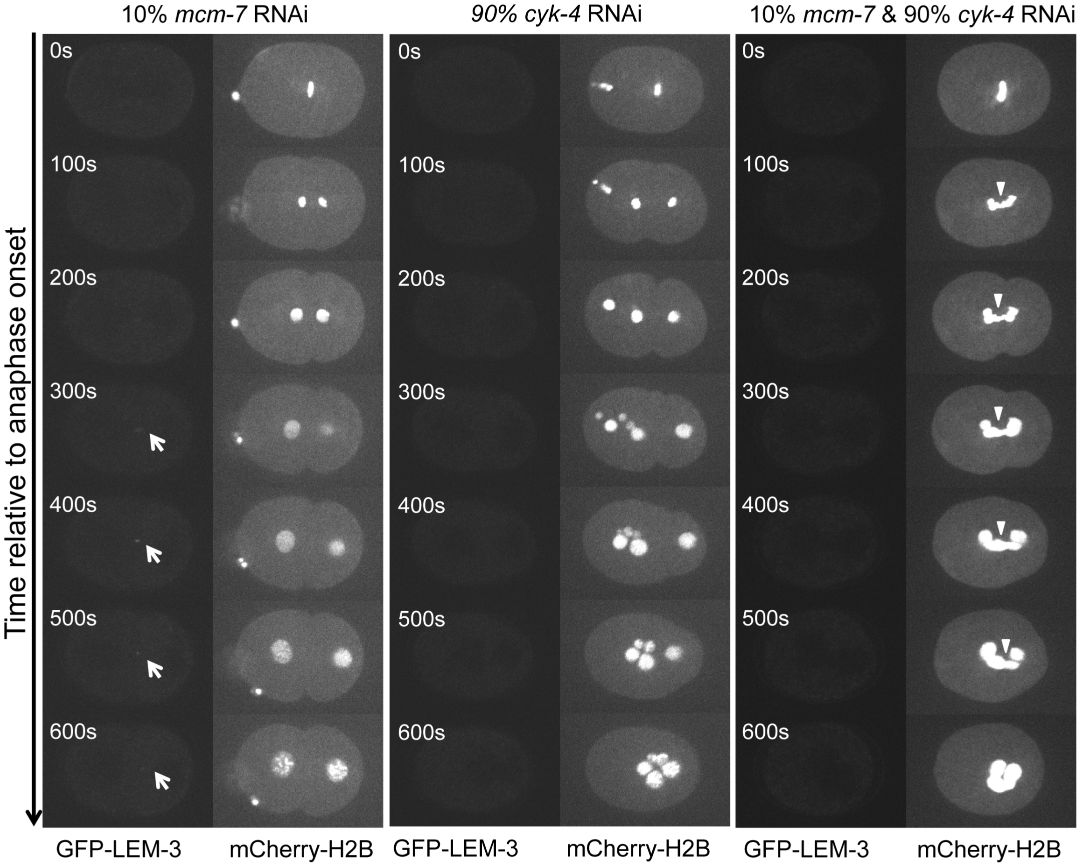

Supplementary Figure 3. The recruitment of LEM-3 to the midbody is important for chromatin bridge resolution. Formation of chromatin bridges upon partial depletion of DNA replication helicase subunit MCM-7 and central spindle component CYK-4. Arrows indicate GFP-LEM-3. Arrowheads indicate chromatin bridges.

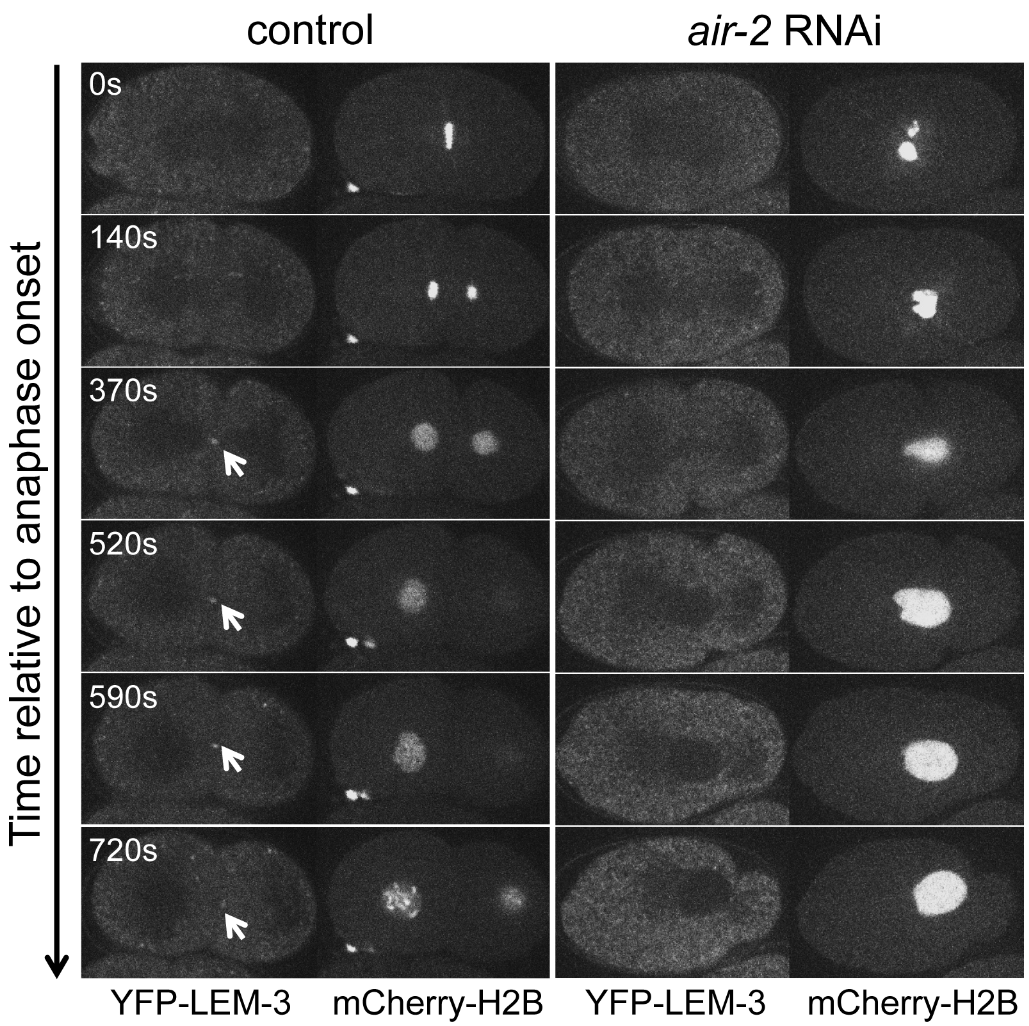

Supplementary Figure 4. LEM-3 localization depends on the AIR-2/Aurora B kinase. LEM-3 foci cannot be detected in *air-2* RNAi embryos. Arrows indicate YFP-LEM-3.
.

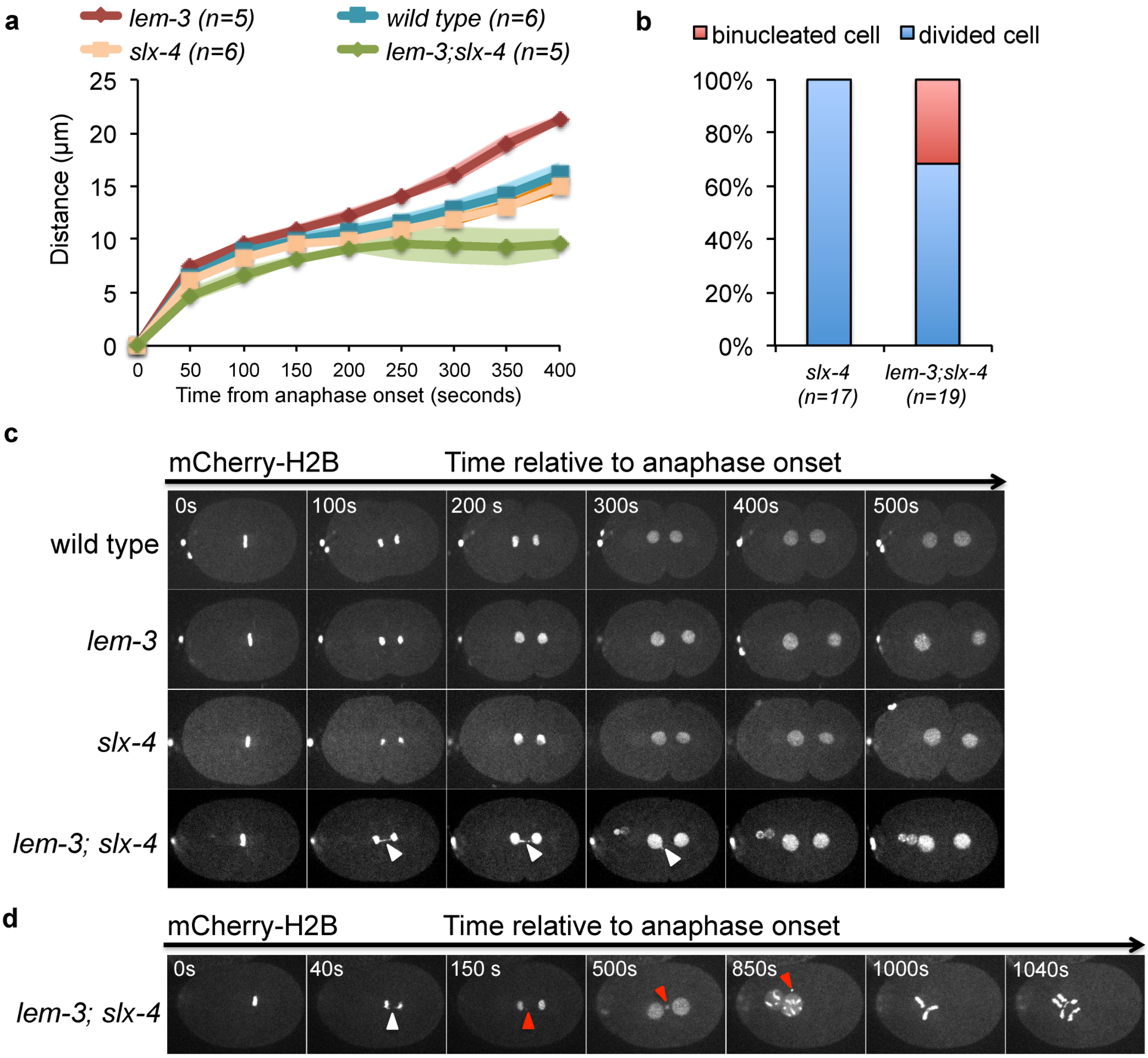

Supplementary Figure 5. Chromosome segregation defects in *lem-3; slx-4* double mutants. (a) Analysis of chromosome segregation in wild type, *lem-3*, *slx-4* single mutants and *lem-3; slx-4* double mutants by measuring the distance between two separated nuclei. The sample size (n) indicates the total number of embryos examined for each genotype. (b) Quantification of binucleated cell formation in *lem-3; slx-4* double mutants. The sample size (n) indicates the total number of embryos examined for each genotype. (c) Chromosome segregation in embryos of indicated genotypes. Representative images were taken from time-lapse recordings of an embryo expressing mCherry-H2B from the anaphase onset of first mitotic division. White arrowheads indicate chromatin bridges. (d) Example of binucleated cells observed in *lem-3; slx-4* double mutants. Images were taken from a time-lapse recording of an embryo expressing mCherry-H2B from the anaphase onset of first mitotic division. White arrowheads indicate chromatin bridges. Red arrowheads indicate micronuclei. The formation of a tripolar spindle is evident ~1000 seconds after the first anaphase.

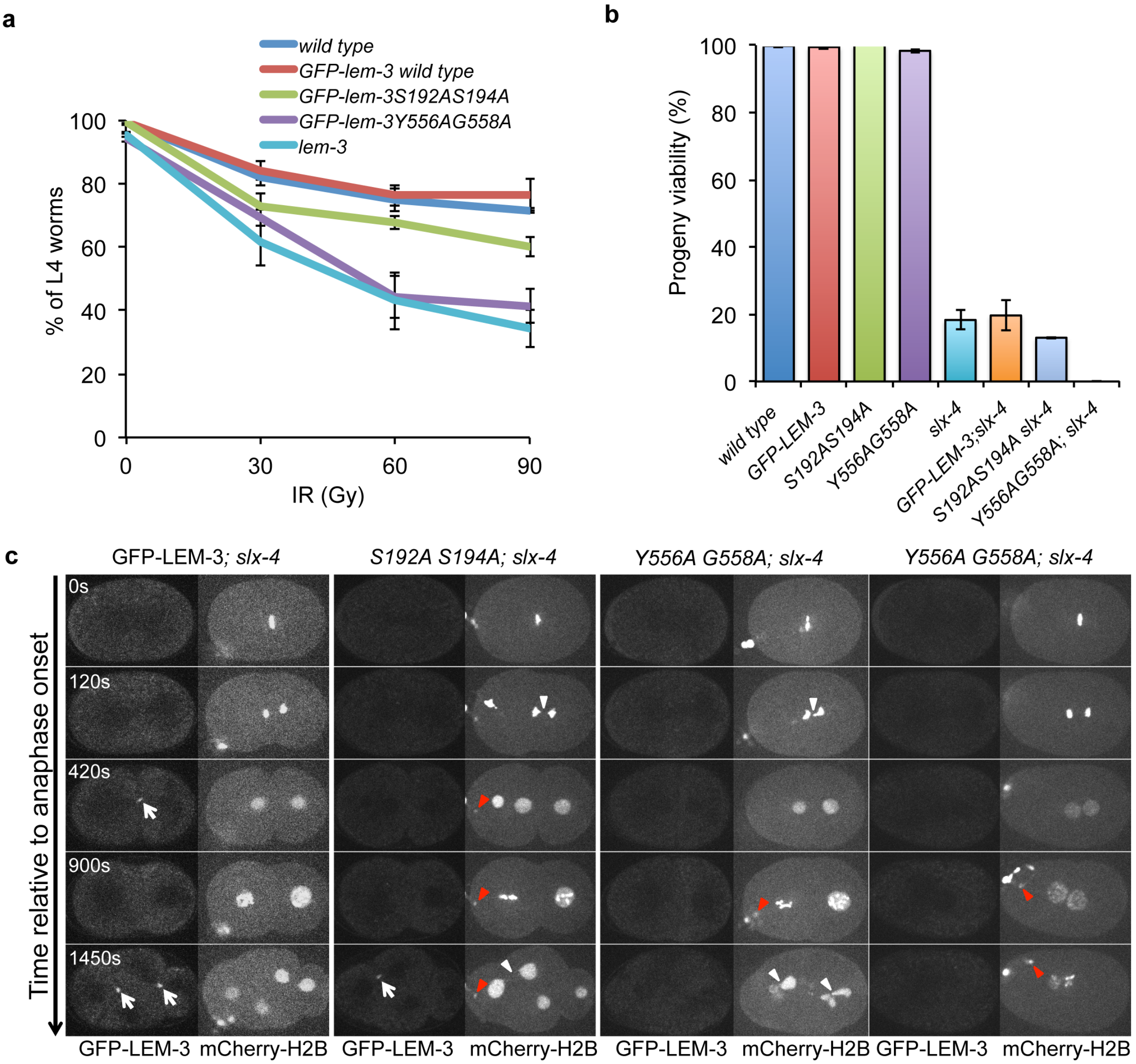

Supplementary Figure 6. Compromised chromosome segregation in *GFP-lem-3 S192AS194A; slx-4* and *GFP-lem-3 Y556AG558A; slx-4* double mutants. (a) Sensitivity of *GFP-lem-3 S192AS194A* and *GFP-lem-3 Y556AG558A* mutants to ionizing radiation (IR). Development delay was scored 48 hours after IR by calculating the percentage of nematodes that reached the L4 stage. (b) Progeny viability of animals of the indicated genotypes. Percentage of survival progeny was determined by counting number of viable eggs/total number of eggs laid. Error bars represent standard deviation of the mean. (c) Chromosome segregation in *GFP-lem-3 S192AS194A; slx-4* and *GFP-lem-3 Y556AG558A; slx-4* double mutants. Images were taken from time-lapse recordings of embryos expressing mCherry-H2B from the anaphase onset of first mitotic division. Arrows indicate GFP-LEM-3. White arrowheads indicate chromatin bridges. Red arrowheads indicate micronuclei.

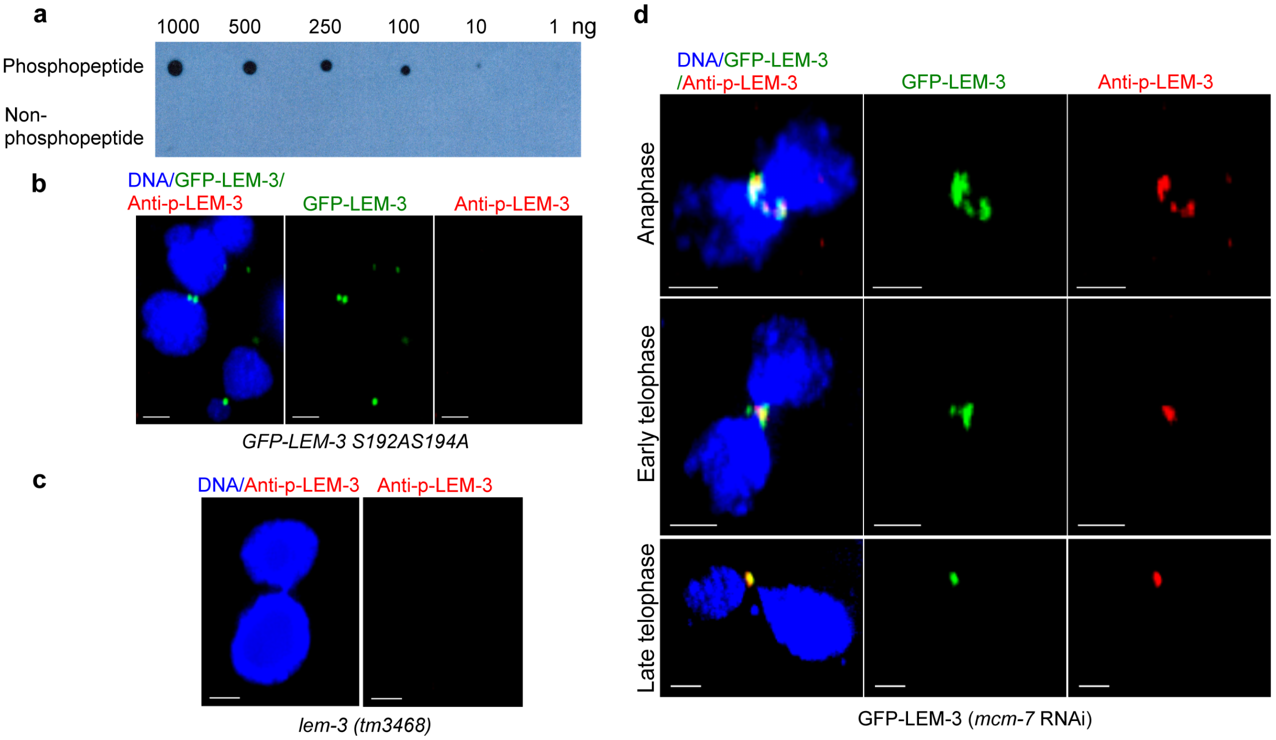

Supplementary Figure 7. Specificity analysis of phospho-LEM-3 antibody. (a) Dot blot analysis to test specificity towards LEM-3 phospho-peptides and non-phospho-peptides. An increasing amount of the phospho- and non-phospho-peptide was spotted on nitrocellulose and probed with a 1:500 dilution of the anti-phospho LEM-3 antibody and a ten-fold dilution of non-phospho-peptide as blocking agent. (b) Immunostaining of phospho-LEM-3 in *GFP-lem-3 S192AS194A* mutant embryos. (c) Immunostaining of phospho-LEM-3 in *lem-3 (tm3468)* embryos. (d) Representative images of GFP-LEM-3 co-localizing with phospho-LEM-3 at midzone /midbody in the presence of chromatin bridges induced by *mcm-7* RNAi. Scale bars: 2 µm.

Supplementary Table 1

List of strains

Strain name	Genotype
SP483	*lem-3 (mn155) I premature stop codon at R190*
TG4254	*lem-3 (tm3468) I 330 bp in frame deletion taking out F141 to V250*
DW102	*brc-1(tm1145) III*
YA665	*lig-4(ok716) III*
TG2534	*polq-1 (tm2026) III*
TG4256	*lem-3 (mn155) I; brc-1(tm1145) III*
TG4258	*lem-3 (mn155) I; lig-4(ok716) III*
TG4307	*lem-3 (mn155) I; polq-1(tm2026) III*
TG1869	*him-18(tm2181)/qC1 III* (consistent with nomenclature in most species we refer to *him-18* as *slx-4*)
TG4259	*lem-3 (mn155) I; him-18(tm2181)/qC1 III*
TG3089	*lem-3 (tm3468) I; him-18(tm2181)/qC1 III*
TG4260	*odIs57[Ppie-1 mCherry::histoneH2B unc-119(+)], unc-119 (ed3)*
4G4261	*lem-3 (mn155) I; odIs57[Ppie-1 mCherry::histoneH2B unc-119(+)], unc-119 (ed3)*
TG4262	*him-18(*tm2181)/qC1 III*; odIs57[Ppie-1 mCherry::histoneH2B unc-119(+)], unc-119 (ed3) note: slx-4 is him-18*
TG4263	*lem-3 (mn155) I; him-18(tm2181)/qC1 III; odIs57[Ppie-1 mCherry::histoneH2B unc-119(+)], unc-119 (ed3)*
TG4264	*opIs383 [Pnpp-1::YFP::lem-3::3'UTRlem-3]*
TG4265	*opIs383 [Pnpp-1::YFP::lem-3::3'UTRlem-3]; odIs57[Ppie-1 mCherry::histoneH2B unc-119(+)], unc-119 (ed3)*
TG4266	*opIs383 [Pnpp-1::YFP::lem-3::3'UTRlem-3] unc-119(ed3) III; HzIs169 [pie-1/mCherry:ZEN-4; unc-119 (+)] ; odIs57[Ppie-1 mCherry::histoneH2B unc-119(+)], unc-119 (ed3)*
TG4267	*cop859 [Plem-3::eGFP::STag:: lem-3::3'UTRlem-3]*
TG4268	*cop859 [Plem-3::eGFP::STag:: lem-3::3'UTRlem-3]; tIs44pAA173; [pie-1p-mCherry::PH(PLC1delta1) + unc-119(+)]; odIs57[Ppie-1 mCherry::histoneH2B unc-119(+)], unc-119 (ed3)*
TG4269	*opIs257 [Prad-54::rad-54::YFP::3'UTRrad-54]*
TG4270	*ltIs38 [pAA1; pie-1/GFP::PH(PLC1F1); unc-119 (+)];odIs57[Ppie-1 mCherry::histoneH2B unc-119(+)], unc-119 (ed3)*
TG4271	*brc-1(tm1145) III; odIs57[Ppie-1 mCherry::histoneH2B unc-119(+)], unc-119 (ed3)*
TH4272	*lem-3 (mn155) I; brc-1(tm1145) III; odIs57[Ppie-1 mCherry::histoneH2B unc-119(+)], unc-119 (ed3)*
TG4273	*cop859 [Plem-3::eGFP::STag:: lem-3 S192AS194A::3'UTRlem-3]; odIs57[Ppie-1 mCherry::histoneH2B unc-119(+)], unc-119 (ed3)*
TG4274	*cop859 [Plem-3::eGFP::STag:: lem-3 Y556AY558A::3'UTRlem-3]; odIs57[Ppie-1 mCherry::histoneH2B unc-119(+)], unc-119 (ed3)*
