# Supplementary material for: LEM-3 is a midbody-tethered DNA nuclease that resolves chromatin bridges during late mitosis
Source: Nat Commun. 2018 Feb 20;9:728. doi: 10.1038/s41467-018-03135-w (PMC5820297; doi:10.1038/s41467-018-03135-w)
Supplement: Supplementary file 3 — Description of Additional Supplementary Files [file 41467_2018_3135_MOESM3_ESM.pdf]

Description of Additional Supplementary Files

File Name: Supplementary Movie 1
Description: Localization of YFP-LEM-3 during cell division.

File Name: Supplementary Movie 2
Description: Co-localization of YFP-LEM-3 and mCherry-ZEN-4 at the midbody.

File Name: Supplementary Movie 3
Description: YFP-LEM-3 localization upon *mcm*-7 RNAi.

File Name: Supplementary Movie 4
Description: Aberrant chromosome segregation in *lem*-3; *brc*-1 mutants upon treatment with IR.

File Name: Supplementary Movie 5
Description: Chromatin bridge resolution by LEM-3.

File Name: Supplementary Movie 6
Description: Chromatin bridge formation upon partial *mcm*-7 and *zen*-4 RNAi.

File Name: Supplementary Movie 7
Description: Chromatin bridge formation upon partial *mcm*-7 and *cyk*-4 RNAi.

File Name: Supplementary Movie 8
Description: Localization of GFP-LEM-3 S192A S194A.

File Name: Supplementary Movie 9
Description: GFP-LEM-3 S192A S194A localization upon *capg*-1 RNAi.

File Name: Supplementary Movie 10
Description: GFP-LEM-3 Y556A G558A localization upon *capg*-1 RNAi.
